# Efficacy of Butyrate to Inhibit Colonic Cancer Cell Growth Is Cell Type-Specific and Apoptosis-Dependent

**DOI:** 10.3390/nu16040529

**Published:** 2024-02-14

**Authors:** Sema Oncel, Bryan D. Safratowich, James E. Lindlauf, Zhenhua Liu, Daniel G. Palmer, Mary Briske-Anderson, Huawei Zeng

**Affiliations:** 1 USDA-ARS Grand Forks Human Nutrition Research Center, Grand Forks, ND 58203, USA; sema.oncel@usda.gov (S.O.); bryan.safratowich@usda.gov (B.D.S.); james.lindlauf@usda.gov (J.E.L.); daniel.palmer@usda.gov (D.G.P.); mary.briske-anderson@usda.gov (M.B.-A.); 2 School of Public Health and Health Sciences, University of Massachusetts, Amherst, MA 01003, USA; zliu@nutrition.umass.edu

**Keywords:** apoptosis, butyrate, cell proliferation, colon cancer, dietary fiber

## Abstract

Increasing dietary fiber consumption is linked to lower colon cancer incidence, and this anticancer effect is tied to elevated levels of short-chain fatty acids (e.g., butyrate) because of the fermentation of fiber by colonic bacteria. While butyrate inhibits cancer cell proliferation, the impact on cancer cell type remains largely unknown. To test the hypothesis that butyrate displays different inhibitory potentials due to cancer cell type, we determined half-maximal inhibitory concentrations (IC_50_) of butyrate in HCT116, HT-29, and Caco-2 human colon cancer cell proliferation at 24, 48, and 72 h. The IC_50_ (mM) butyrate concentrations of HCT116, HT-29, and Caco-2 cells were [24 h, 1.14; 48 h, 0.83; 72 h, 0.86], [24 h, N/D; 48 h, 2.42; 72 h, 2.15], and [24 h, N/D; 48 h, N/D; 72 h, 2.15], respectively. At the molecular level, phosphorylated ERK1/2 and c-Myc survival signals were decreased by (>30%) in HCT116, HT-29, and Caco-2 cells treated with 4 mM butyrate. Conversely, butyrate displayed a stronger potential (>1-fold) for inducing apoptosis and nuclear p21 tumor suppressor in HCT116 cells compared to HT-29 and Caco-2 cells. Moreover, survival analysis demonstrated that a cohort with high p21 gene expression in their colon tissue significantly increased survival time compared to a low-p21-expression cohort of colon cancer patients. Collectively, the inhibitory efficacy of butyrate is cell type-specific and apoptosis-dependent.

## 1. Introduction

Colorectal cancer (CRC) is a serious health concern in both men and women worldwide. In 2023, it has been predicted that about 153,020 persons will be diagnosed with CRC and 52,550 individuals will die because of the disease in the United States [1]. The development of cancer involves a complex interplay between genetic and environmental factors. Genetic mutations can predispose individuals to cancer (5–10%), but environmental factors (90–95%), such as diet, smoking, infections, and other lifestyle factors also play significant roles [2]. Indeed, colon cancer is highly associated with dietary factors since they can influence the composition of the gut microbiome, which is of critical importance to gut health and subsequent colon health [3]. Daily dietary choices are directly related to colon cancer prevention. For example, consuming a fiber-rich diet (e.g., >38 g/day, a US adult male) significantly decreases colon cancer incidence in humans, while wheat bran fiber inhibits colon tumorigenesis in animal models [4,5,6]. Dietary fiber can be different in chemical composition, and it accounts for a variety of non-digestible food components such as non-starch polysaccharides, cellulose, resistant starch, inulin, and oligosaccharides with associated anti-cancer potential and other health benefits [7,8]. Mechanistically, these health-promoting properties are associated with short-chain fatty acids (SCFAs), and these SCFAs are generated through the fermentation of dietary fiber by bacteria in the colon [4,5,9]. 

The concentrations of SCFAs (including acetate, propionate, and butyrate) in the colon could exceed 100 mM in humans consuming fiber-rich diets (e.g., >40 g/day) [10,11,12]. Interestingly, the SCFA concentrations are not the same in different colon segments in which a SCFA concentration gradient occurs along the villus-to-crypt axis in a human colon [10,11,12]. The colonic surface epithelium consists of differentiated cells such as enterocytes and enteroendocrine cells, while undifferentiated stem-crypt cells divide and migrate to the top of the villi to form more specialized cells through differentiation [13].

Unlike terminally differentiated colonic cells, stem cells and cancer cells are still capable of entering the cell cycle, mitosis, proliferation, and apoptosis processes [14,15,16]. Although SCFAs are important health-promoting bacterial metabolites in the colon, the efficacy of butyrate’s anti-cancer potential remains to be determined in the context of heterogeneous cancer cells. Our recent data demonstrate that, in a single cancer cell type experiment, the efficacy of SCFAs to inhibit cancer cell proliferation is butyrate > propionate > acetate [17]. Bacterial metabolites such as SCFAs not only serve as energy sources for colonic cells but also directly alter cellular signaling activities [16]. These signaling pathways play a critical role in the “decision” of cell death and survival. Thus, we speculate that butyrate-related molecular actions are likely to be colon cell type-specific. Colonic epithelial cancer cell lines (e.g., HCT116, HT-29, and Caco-2 cells) commonly serve as valuable tools to examine the functional aspects of dietary factors on colon cancer prevention [17,18]. There are 3177 and 416 gene mutations in the HCT116 and HT29 cell genomes, respectively, with the primary dysregulation of Kirsten rat sarcoma virus (KRAS) pathway. In contrast, there are only 167 gene mutations in the Caco2 cell genome with the primary dysregulation of Wingless integration site (WNT) pathway [19]. Moreover, colon cancers display histological and molecular differences because of distinct cancer cell types with unique functional characteristics such as the capacity to differentiate [20,21,22]. The differentiating capacity of cancer cells is inversely associated with the aggressiveness of the cancer [23,24].

There are scant data examining the comparative efficacy of butyrate against different cancer cell types. Investigating the impact of butyrate requires careful consideration of the distinct molecular profiles and functions inherent to different types of colon cancer cells. Cellular characteristics such as the differentiating capacity; genetic makeup such as the number of mutation events and percentage copy number altered chromosome regions; or signaling protein expressions in different colon cancer cell types could all contribute to variations in the efficacy of butyrate on these cell lines [25]. We hypothesized that butyrate exhibits different inhibitory efficacies because of colon cancer cell type. The differentiating capacity of colon cancer cells is the greatest in Caco-2 cells, followed by HT-29 cells, and HCT116 cells [20,21,22]. Therefore, we focused on the comparative efficacy of butyrate against these three cell lines at the molecular level in this report. 

## 2. Materials and Methods

### 2.1. Materials, Cell Culture, and Cell Proliferation Assay

Materials, cell culture, and proliferation assays were conducted as previously described [17] with minor modifications. Briefly, we purchased (1) HCT116, HT-29, and Caco-2 colonic cancer cells from American Type Culture Collection, and these cells were maintained/treated in DMEM (Invitrogen, Carlsbad, CA, USA) containing 10% fetal bovine serum (FBS); (2) sodium butyrate (as a source of butyrate) and propidium iodide (PI) (25 μg/mL) from Sigma Chemical Corporation (St. Louis, MO, USA). Stock cells were passaged two times per week at ~80% confluency in Hanks’ cell culture grade buffer with 0.25% trypsin and 1 mM ethylenediamine tetra-acetic acid. Cell proliferation was evaluated using the Beckman Vi-CELL XR Cell counter (Indianapolis, IN, USA) [26]. All cells were cultured at 36.5 °C with 5% CO_2_ at 90–95% humidity in a standard cell incubator, and were free of mycoplasma [27]. 

### 2.2. Cell Apoptosis Analysis

The apoptosis assay procedure was conducted as previously described [17] with minor modifications. Briefly, cell apoptosis was determined using a Guava Nexin^TM^ Kit (Cytek Biosciences, Fremont, CA, USA) which utilizes Annexin V/7-AAD staining. Cells treated with butyrate for 24 h and 48 h in 6-well plates were seeded as follows: HCT116 cells were seeded 300,000 cells/well and 160,000 cells/well; HT-29 cells were seeded 450,000 cells/well and 336,000 cells/well; and Caco-2 cells were seeded 250,000 cells/well and 160,000 cells/well, respectively. HCT116, HT-29, and Caco-2 cells underwent trypsinization and were subsequently suspended in growth media. At least 2000 cell events for each sample were examined for apoptosis by the Guava EasyCyte 6HT flow cytometry analyzer with GuavaSoft 3.3 (Cytek Biosciences, Fremont, CA, USA). 

### 2.3. Western Blotting Analysis

Cell lysates for Western blotting were prepared as previously described [17] with minor modifications. Briefly, HCT116, HT-29, and Caco-2 cells in 60 mm culture dishes were treated with butyrate (0 to 4 mM) when cells reached ~30–40% confluency. Cells were harvested and lysed 24 h after butyrate treatment using cell lysis buffer (Cell Signaling Technology, Inc., Danvers, MA, USA). Bradford assay was performed to determine the protein concentration. Protein samples were separated with SDS-page gel electrophoresis using gradient gels (4 to 20%) and transferred onto PVDF membranes. After blocking the membranes with 5% dry milk, membranes were incubated overnight at 4 °C with the following primary antibodies: phospho-p44-42 (ERK1/2) T-202/Y-204, ERK1/2, p21, glyceraldehyde-3-phosphate dehydrogenase (GAPDH) (Cell Signaling Technology, Inc., Danvers, MA, USA), and c-Myc (Abcam, Cambridge, MA, USA). After TBS wash, membranes were incubated for 1 h with anti-rabbit HRP-conjugated secondary antibody (1:5000 dilution) (Cell Signaling Technology, Inc., Danvers, MA, USA). Finally, these membranes were TBS-washed and then incubated with chemiluminescence (ECL) reagent; and protein images were visualized and quantified using a LI-COR Odyssey Fc imager system (Lincoln, NE, USA).

### 2.4. Immunofluorescent Staining

Immunofluorescent staining was performed as previously described [17] with minor modifications. Briefly, HCT116, HT-29, and Caco-2 cells were treated with butyrate (0 to 4 mM) for 24 h when they reached 30–40% confluency on chambered microscope slides. Subsequently, these cells were fixed in 4% paraformaldehyde for 15 min (at the end of butyrate treatment), which was then immediately followed by 10 min permeabilization at −20 °C with 100% methanol. After a rinse with PBS, cells were blocked for 1 h with goat serum (5%) and then incubated overnight with p21 antibody at 4 °C (to promote epitope/antibody binding). After PBS wash and (1 h) incubation with anti-rabbit Immunoglobulin G conjugated Alexa Fluor^®^ 488 (Cell Signaling Technology, Inc., Danvers, MA, USA), the coverslips were mounted onto cells on chamber slides using fluoroshield-mounting media containing PI (25 μg/mL). Images (~2000 cells/treatment) were taken with a Nikon E400 microscope and quantified by Image Pro Plus version 9.1 (Media Cybernetics, Inc., Rockville, MD, USA). 

### 2.5. Analysis p21 Gene Expression in Human Tumor Tissues and Survival Analysis 

Two distinct online tools were utilized for conducting gene expression and survival analysis in this study. (A) The CDKN1A (p21) expression in human tumor tissues: we examined the Gene Expression database of Normal and Tumor tissues 2 (*GENT2*) (http://gent2.appex.kr/gent2/ (accessed on 7 September 2023) in the five most common cancer types (breast, prostate, lung, colon, and skin cancer) in the United States [28]; and (B), p21 gene expression and colon cancer patient survival, which we examined the Human Protein Atlas dataset (http://www.proteinatlas.org (accessed on 28 August 2023) of gene expression in cohorts of patients with colon adenocarcinoma.

### 2.6. Statistical Analysis

Statistical analysis was performed as previously described [17] with minor modifications. Briefly, Data are shown as means ± standard deviations (SDs). As cell counting data had high variability, we log-transformed data to fit a linear model based on the diagnostic plots of the residuals. A three-parameter logistic model to percent inhibition was fit to predict the concentration of butyrate required for 50% cell growth inhibition (IC_50_) using log concentration as a predictor variable and count as the dependent variable. PROC NLIN, a fitting modeling procedure in SAS, was employed for model fitting (SAS Institute, Inc., Cary, NC, USA) [29]. We performed cell proliferation assays individually for each cell line because a simultaneous assay of all three cell lines would generate too many samples for accurate analysis within a given time frame. Subsequently, one-way analysis of variance (ANOVA) was used to test the equality of the concentration group means for cell proliferation, apoptosis, Western blotting, and immunofluorescent staining data. Tukey’s honestly significant difference (HSD) tests were used for pairwise multiple comparisons. Kaplan–Meier survival curve analysis was applied to p21 gene expression data, and log-rank tests compared survival curves in two distinct p21 expression colon cancer patient cohorts. Two sample *t*-tests assuming unequal variances were performed for each tissue type to compare the expressions between cohorts. For each analysis, four independent experiments (*n* = 4) were repeated, and all other analyses were performed using JMP V 17 software (SAS Institute, Inc., Cary, NC, USA), with a significance level set at α = 0.05.

## 3. Results

### 3.1. Distinct Effects of Butyrate on Cell Proliferation among Three Cell Types

**Figure 1:**
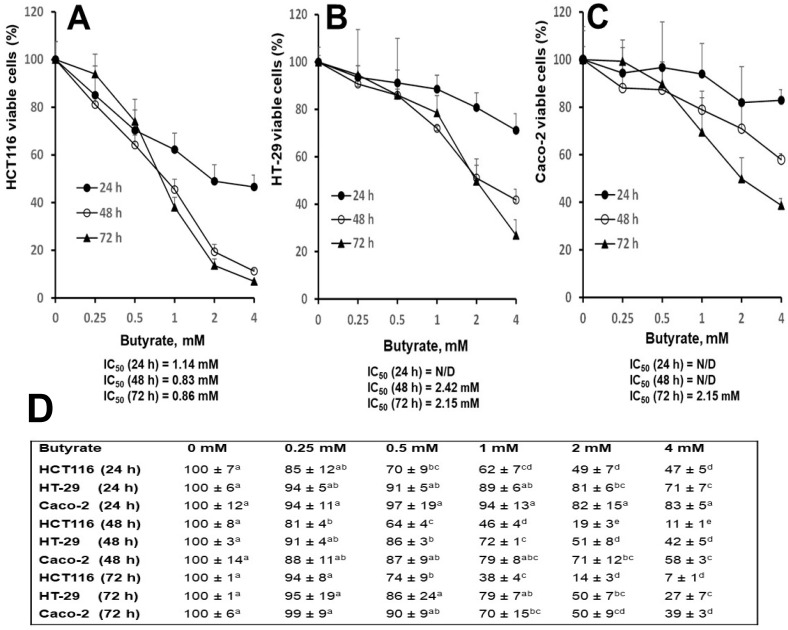
Distinct effects of butyrate on colon cancer cell lines (**A**) HCT116, (**B**) HT-29, (**C**) Caco-2 cell growth curves for 24, 48, and 72 h, (**D**) data for cell growth curve (**A**–**C**). The “N/D” stands for “not detectable”. Letters that are not shared between concentration group means are significantly different by row within cell lines using Tukey’s HSD after on-way ANOVA, *p* < 0.05, at a given time point, cell growth curves were repeated (*n* = 4) for each cell line, and data are presented as means ± SDs.

To determine inhibitory efficacy, IC_50_ butyrate-concentrations were determined utilizing HCT116, HT-29, and Caco-2 cell growth curves. Cells cultured on six-well plates were 25% to 40% confluent at the beginning of treatment, followed by 80% to 90% (if 0 mM butyrate) confluency at the end of treatment. At 24, 48, and 72 h time points, IC_50_ (mM) concentrations of butyrate were [1.14, 0.83, and 0.86], [N/D, 2.42, and 2.15], and [N/D, N/D, and 2.15] in HCT116, HT-29, and Caco-2, respectively. While the IC_50_ values of all three-time points could be calculated for HCT116 cells, only the IC_50_ values of HT-29 at 48 and 72 h and Caco-2 at 72 h could be calculated (Figure 1A–C). At 72 h, the IC_50_ butyrate-concentrations in HT-29 and Caco-2 cells were more than 1-fold greater than that of HCT116 cells. 

Consistent with the above observation, at 24 h, cell numbers were decreased in both HCT116 cells (51%, and 53%) and HT-29 cells (19%, and 29%) but not in Caco-2 cells with 2 and 4 mM butyrate treatments, compared to untreated cells, respectively (Figure 1D). Similarly, at 48 h, the cell number was decreased in HCT116 cells (81%, and 89%), HT-29 cells (49%, and 58%), and Caco-2 cells (29%, and 42%), following 2 and 4 mM butyrate treatment, compared to untreated cells, respectively (Figure 1D); at 72 h, the cell number was decreased in HCT116 cells (86%, and 93%), HT-29 cells (50%, and 73%), and Caco-2 cells (50%, and 61%) treated with 2 and 4 mM butyrate, compared to untreated cells, respectively (Figure 1D).

### 3.2. Distinct Effects of Butyrate on Apoptosis among Three Cell Types

**Figure 2:**
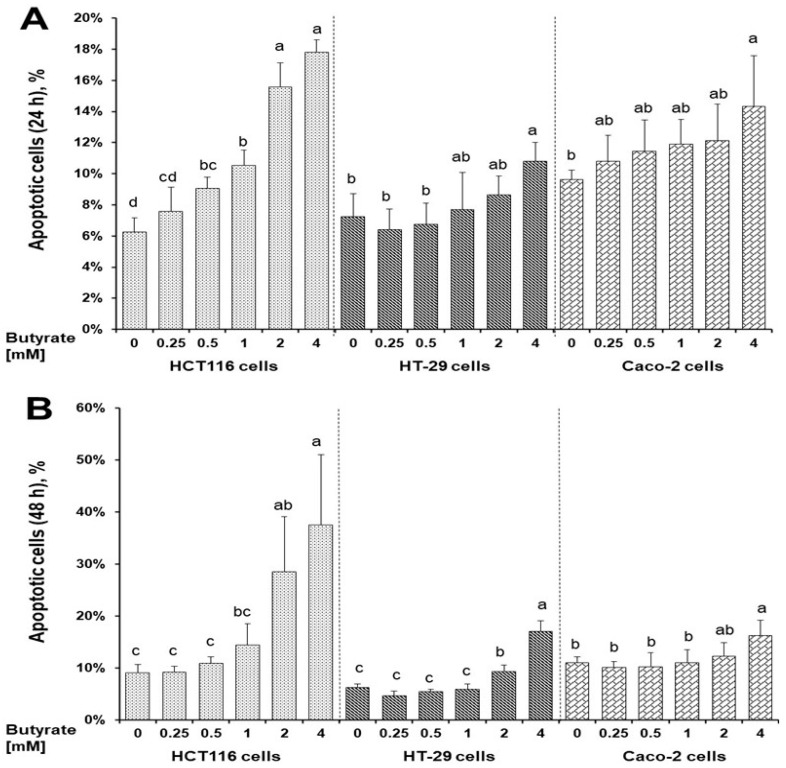
Distinct effects of butyrate on apoptosis in HCT116, HT-29, and Caco-2 cells for (**A**) 24 h, (**B**) 48 h. Data are presented as means ± SDs for each group within each cell line at 24 and 48 h (*n* = 4). Means that do not share the same letters within a cell line are significantly different using Tukey’s HSD following one-way ANOVA, *p* < 0.05.

The number of apoptotic cells exhibited a dose-dependent increase with 0.7-, 1.5-, and 1.9-fold changes at 24 h, respectively, following 1, 2, or 4 mM butyrate treatment compared to untreated HCT116 cells (Figure 2A and Figure S1). In contrast, when treated with 4 mM butyrate at 24 h, apoptotic cells increased 0.5-fold in HT-29 and Caco-2 cells compared to that of their respective untreated cells (Figure 2A and Figure S1). Correspondingly, at 48 h, the butyrate dose-dependent apoptosis effect was stronger in HCT116 cells than that of HT-29 and Caco-2 cells; and a maximum increase of 3.1-, 1.7-, and 0.5-fold in HCT116, HT-29 and Caco-2 cells (treated with 4 mM butyrate) compared to their respective untreated cells (Figure 2B and Figure S1).

### 3.3. Distinct Effects of Butyrate on Signaling Proteins among Three Cell Types 

**Figure 3:**
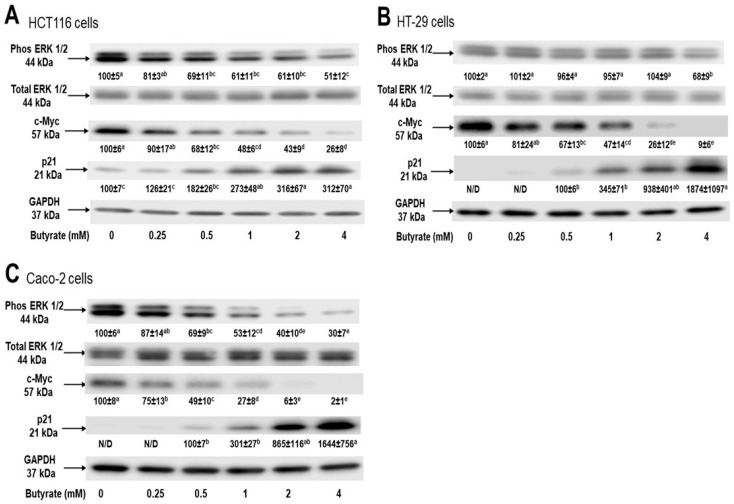
Distinct effects of butyrate on colon cancer cell signaling, (**A**) HCT116, (**B**) HT-29, (**C**) Caco-2 for 24 h via Western blot analyses. Data are presented as means ± SDs for each concentration group within each cell line for signaling pathways (*n* = 4). The “N/D” stands for “not detectable”. Letters that are not shared between concentration group means are significantly different by row within cell lines using Tukey’s HSD after one-way ANOVA, *p* < 0.05.

To examine the key cellular signaling molecules accounting for these distinct effects of butyrate on inhibiting cancer cell growth, we investigated phosphorylated ERK1/2, c-Myc, and p21 protein levels in these three different cell lines. We examined these signaling proteins at the earliest time point for apoptosis analysis at 24 h, to reduce bystander gene-effect at 48 h time point. The phosphorylated ERK1/2 protein levels were decreased by (39%, 39%, and 49%) and (47%, 60%, and 70%) in HCT116 and Caco-2 cells, respectively, following 1, 2, or 4 mM butyrate treatment, compared to untreated cells, respectively. However, only a 32% decrease in the phosphorylated ERK1/2 protein level was detected in HT-29 cells following treatment with 4 mM butyrate. Similarly, in a dose-dependent manner, the level of c-Myc protein was decreased by 74%, 91%, and 98% in HCT116, HT-29, and Caco-2 cells treated with 4 mM butyrate, respectively. In contrast, the p21 protein level was increased by >2-, 17-, and 15-fold in HCT116, HT-29, and Caco-2 cells, following 4 mM butyrate treatment, respectively (Figure 3).

### 3.4. Distinct Effects of Butyrate on p21 Protein’s Subcellular Localization among Three Cell Types

**Figure 4:**
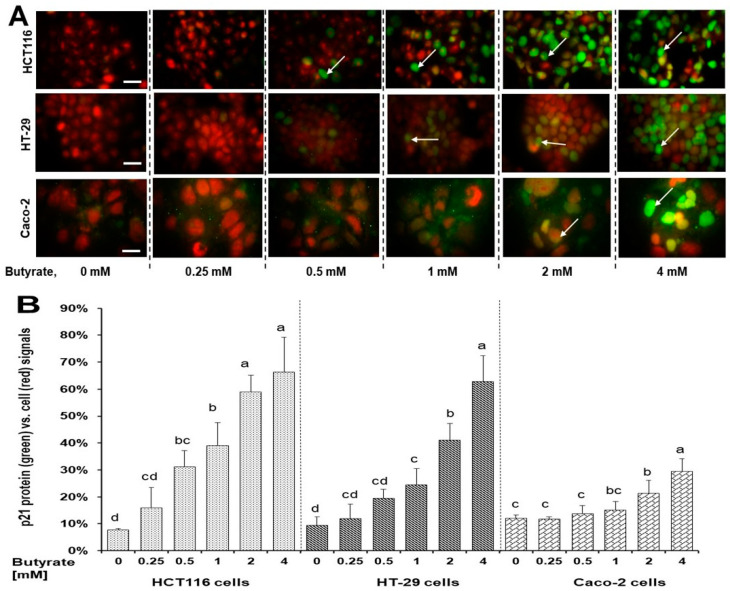
Distinct effects of butyrate on the level of p21 protein and cellular distribution in HCT116, HT-29, and Caco-2 colon cells for 24 h. (**A**) Each (merged) image consists of two original images: image 1, cells were treated with anti-p21 antibody, and conjugated anti-Rabbit IgG (green signals); image 2, cells were mounted using fluoroshield containing PI as counter staining for cell background specifically nuclei (red signals) at 1000× magnification. Scale bars (25 μm) were embedded into the lower-right corner of each (0 mM) image, white arrows indicated the intense p21 protein at the nucleus; (**B**) The percentage ratio of p21 protein signal vs. background cellular signals. Data are presented as means ± SDs for percentage ratio of p21 protein signal vs. background cellular signals by concentration group within each cell line (*n* = 4). Concentration group means that do not share a common letter are significantly different within cell lines by using Tukey’s HSD after performing one-way ANOVA, *p* < 0.05.

Both p21 protein and its subcellular localization are crucial to regulate cell proliferation [30]. We found that the butyrate increased p21 protein content and its nuclear localization (Figure 4A and Figure S2). In a dose-dependent manner, the ratio percentage of p21 protein level to the overall cell background at 24 h was (39%, 59%, and 66%), (24%, 41%, and 62%), and (15%, 21%, and 30%) in HCT116, HT-29 cells and Caco-2 cells, following 1, 2, and 4 mM butyrate treatment, compared to untreated cells, respectively (Figure 4B). 

### 3.5. High and Low p21 Expression Cohorts Differ in Survival Time after Diagnosis with Colon Cancer

**Figure 5:**
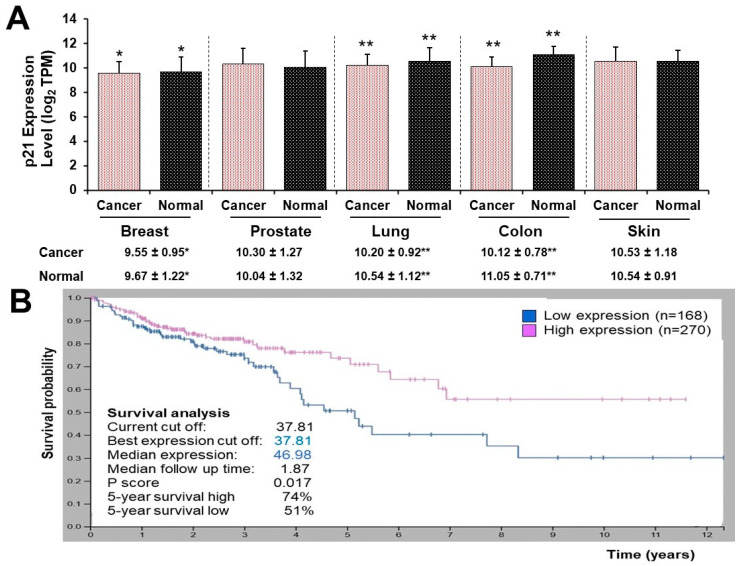
Gene expression of p21 (**A**) in the 5 most common cancer types. Breast tissues (cancer, *n* = 5574 vs. normal, *n* = 475); Prostate tissues (cancer, *n* = 297 vs. normal, *n* = 56); Lung tissues (cancer, *n* = 2362 vs. normal, *n* = 508); Colon tissues (cancer, *n* = 3774 vs. normal *n* = 396); Skin tissues (cancer, *n* = 547 vs. normal, *n* = 263). (**B**) in cohorts of patients with colon adenocarcinoma. Survival analysis shows that p21 mRNA level significantly differs between the high and low groups in survival time after diagnosis in colon cancer patients [low expression, *n* = 168 (blue curve) vs. high expression (red curve), *n* = 270, *p* = 0.017]. Means of p21 gene expression (mRNA values) differ, * *p* < 0.05, and ** *p* < 0.0001 by Student *t*-tests.

The p21 mRNA levels of breast, lung, and colon cancer types were decreased when compared to their respective normal tissues. The p21 level of colon cancer tissue was the lowest (cancer, 10.122 vs. normal, 11.050), followed by lung cancer (cancer, 10.204 vs. normal 10.543) and breast cancer (cancer, 9.550 vs. normal 9.667) with log_2_ (transcripts per million, TPM). However, the p21 mRNA level of prostate and skin cancer tissues did not differ when compared to their normal tissues (Figure 5A).

Survival analysis of the Human Protein Atlas cancer dataset demonstrated that high and low p21 gene expression cohorts significantly differed in survival time after diagnosis with colon cancer (*p* = 0.017). For a 5-year survival time after diagnosis, the high p21 expression cancer cohort was 23% greater than that of the low p21 expression cancer cohort. Fragments per kilobase of exon model per million reads mapped (FPKM) value of the p21 gene, resulting in the most significant survival difference, serve as the threshold to distinguish between the two cohorts (Figure 5B). 

## 4. Discussion

During the past two decades, a growing body of research evidence reveals that most tumors and cancers are intricate ecosystems evolved from their original tissue microenvironments [31]. With recent next-generation sequencing, a high number of mutations have been characterized in colon cancer, and cancer (inter-patient and intra-tumor) heterogeneity has been well recognized. This heterogeneity consists of various cell types with different gene expression profiles [32]. Thus, intra-tumor heterogeneity leads to a tumor with a variety of biological properties that can contribute to drug resistance, recurrence and metastasis [32]. Accumulating data have shown butyrate’s anticancer potential [16], and this inhibitory effect on cancer cells may be predominantly mediated by butyrate’s histone deacetylase inhibitor activity [33,34]. Moreover, recent data indicate that butyrate modulates cell cycle, apoptosis, and formation of reactive oxygen species (ROS) in colon cells [35,36], critical mechanistic events for cell proliferation. While substantive evidence demonstrates that butyrate inhibits colon cancer cells via several molecular mechanisms [16,37], there are scant data examining the inhibitory efficacy of butyrate against colon cancer cell proliferation in the context of cancer cell types related to intra-tumor heterogeneity. To address this gap, we meticulously selected diverse colon cell lines based on the gene mutation rate of cell genomes, differentiating capacity, and cancer cell aggressiveness.

In this report, the efficacy of butyrate to inhibit colon cell growth was the greatest in HCT116 cells followed by HT-29 cells and Caco-2 cells based on IC_50_ values (Figure 1). Similarly, the potential of butyrate to induce cell apoptosis was the greatest in HCT116 cells followed by HT-29 cells and Caco-2 cells (Figure 2). These observations are consistent with that butyrate inhibits colon cancer cell proliferation, and cell apoptosis plays an essential role during this process [16]. Though all are human colon cancer cell lines, there are distinct variations in cell characteristics among HCT116, HT-29, and Caco-2 cells. Notably, (1) the gene mutation rate of cell genomes is “HCT116 cells > HT-29 cells > Caco-2 cells” [19]; (2) Caco-2 cells (but not HT-29 and HCT116 cells) are uniquely able to differentiate into a monolayer of cells with many properties typical of absorptive enterocytes [20,21,22,23,24]; (3) HT-29 cells (but not HCT116 cells) feature enterocyte microvillus, a functional adaptation for nutrient absorption [20,21,22,23,24]. In terms of tumor formation and metastasis in immunocompromised mice, HCT116 cells show greater potential compared to HT-29 and Caco-2 cells [20,21,22,23,24]. The inverse relationship between differentiating capacity (Caco-2 cells > HT-29 cells > HCT116 cells) [20,21,22] and cancer aggressiveness/gene mutations [23,24] implies that butyrate may have a stronger inhibitory potential against aggressive cancer cells than “less” aggressive cancer cells in the colon, as suggested by, our inhibitory data (Figure 1). These data also suggest butyrate’s potential impact on critical biological aspects of cancer biology. (1) High cell proliferation rates and subsequent rapid migration and metastasis are characteristics of aggressive cancer cells in the colon [32] and the leading causes of mortality of colon cancer patients. Increased butyrate concentrations may greatly mitigate these high cell proliferation rates, migration, and metastasis [38]. (2) Because surgical approaches can only eliminate the primary lesion and tumor, increasing butyrate concentration in the colon may provide an additional valuable means (other than drug treatment) to reduce cancer recurrence in colon cancer patients. (3) As a step to verify these theories, future human studies are warranted to determine if there is an inverse association between the content of human colonic butyrate (e.g., fecal butyrate) and cancer metastasis, recurrence, and subsequent mortality rates after surgical procedures in colon cancer patients.

Another important feature of cancer biology is the existence of intra-tumor heterogeneity which constitutes diverse cancer cell types [32]. Thus, the impact of colon cancer cell type on butyrate’s cancer cell inhibition is crucial in this regard. The efficacies of butyrate to inhibit cancer cell growth (Figure 1), and to induce cancer cell apoptosis (Figure 2), show a positive correlation within the context of cell type specificity, with “HCT116 cells > HT-29 cells > Caco-2 cells”. These data suggest that (a) the efficacy of butyrate to inhibit cancer cell growth is cell type-specific, and (b) apoptosis is a key cellular action underlying butyrate’s anticancer cell activity. At the molecular level, extracellular signal-regulated kinase ½ (ERK1/2) is critical to cell proliferation signaling and generally activated (phosphorylated) in response to survival signals that counteract apoptotic stimuli [39,40]. Dysregulation of the ERK1/2 pathway may contribute to tumorigenesis through promoting tumor cell proliferation and invasion [41]. Mutations in genes involved in the ERK1/2 pathway occur in many different cancer tissues, and oncogene-targeted therapies that directly inhibit ERK1/2 signaling can trigger tumor cell death [41]. Within this research, butyrate inhibited the phosphorylation of ERK1/2 in all three colon cancer cell lines (Figure 3), which suggests that butyrate may reduce survival signaling in colon cancer cells and increase subsequent cell apoptosis (Figure 2). While the efficacy of butyrate to inhibit ERK1/2 activation was greater in Caco-2 cells than in HCT116 cells and HT29 cells (Figure 3), the efficacy of butyrate (4 mM) to cause apoptosis in HCT116 cells was much greater (e.g., 1.9-fold at 24 h) when compared to that in HT-29 cells and Caco-2 cells (e.g., 0.5-fold at 24 h) (Figure 2). This observation indicates that inhibition of ERK1/2 activation alone is not directly associated with the inhibition of cancer cell proliferation. This suggests that the apoptosis is likely to be the combined effects of ERK1/2 on downstream genes and other related signaling pathways. 

Along the same line, recent data demonstrate that myelocytomatosis oncogene (c-Myc) is a downstream target gene of the ERK1/2 signaling pathway, and disruption of the ERK1/2-c-Myc signaling pathway within tumor endothelial cells is sufficient to halt tumor enlargement [42]. The proto-oncogene c-Myc plays a pivotal role as a major regulator of cellular proliferation involved in metabolic and apoptotic action in cancer cells, and it is overexpressed in numerous types of cancer including colonic carcinomas [43,44]. Our data reveal a significant inhibition of c-Myc protein levels by butyrate (Figure 3), aligning with the understanding that c-Myc is a downstream gene of the ERK1/2 pathway [42]. These findings indicate that butyrate inhibits the ERK1/2-c-Myc pathway in colon cancer cells, which may be the key mechanistic action to protect against colon cancer. However, the efficacy of butyrate (4 mM) to reduce c-Myc protein levels is slightly greater in HT-29 cells (91%) and Caco-2 cells (98%) than in HCT116 cells (78%) (Figure 3). These data suggest that c-Myc protein reduction alone is not directly associated with butyrate-induced apoptosis (Figure 2). Thus, cell growth inhibition (Figure 1) may be the combined effects of c-Myc downstream genes and other relevant cell signaling cascades. 

The p21 gene is a putative tumor suppressor, also known as cyclin-dependent kinase inhibitor 1A (CDKN1A) [45,46]. The protein level of p21 is often decreased in cancer tissues [47] and is repressed by c-Myc at the transcriptional and post-transcriptional levels via activating multiple pathways [45,46]. Our data showed a reduction in c-Myc protein level but an elevation in p21 protein levels upon butyrate treatment in all three cell lines (Figure 3), which may mediate cancer cell apoptosis [48,49]. While the p21 protein may be involved in anticancer signaling pathways, its function depends on intra-cellular localization [48,50]. Although nuclear p21 serves as a negative regulator of cell growth, cytoplasmic p21 may promote cell proliferation and impede apoptosis under certain conditions [48,50]. In addition to the increase in p21 protein levels in whole cells (Figure 3), the immunofluorescent staining analysis revealed that, upon butyrate treatment, the predominant localization of p21 protein was around or within the cell nuclei of HCT116, HT-29, and Caco-2 cells (Figure 4A). Compared to the untreated cells, the increase in the percent ratio of nuclear p21 was the greatest in HCT116 cells followed by HT-29 cells, and then Caco-2 cells (Figure 4B). Thus, the efficacy of butyrate to increase the percent ratio of nuclear p21 (Figure 4) is positively associated with the efficacies of butyrate to inhibit cell proliferation (and to increase apoptosis) in HCT116 cells, HT-29 cells, and Caco-2 cells. The p21 gene, a c-Myc downstream gene, is essential to directly regulate mitosis during cell proliferation [45,46]. Consequently, these findings strongly suggest that the ERK1/2-c-Myc pathway leads to nuclear localization of the p21 protein, a suppressor of cell growth, and plays a pivotal direct role in butyrate’s inhibitory efficacy against colon cancer proliferation.

To combine the above novel mechanistic insights and human clinical data, we analyzed the p21 protein in GENT2 gene expression profiles in a cohort of cancer patients (top five most common cancer types in the United States [28]) compared to their respective normal control tissues (Figure 5A). Our finding showed that the p21 mRNA level was decreased to the greatest extent in colon cancer among the top five common cancer types (Figure 5A), suggesting that p21 gene expression may play the biggest inhibitory effect on colonic tumorigenesis compared with other cancer types. The fact that the colon cancer cohort with high p21 expression exhibited a 23% higher 5-year survival rate compared to the low p21 expression cohort (Figure 5B) further strengthens the potential protective role of p21 protein against colon cancer.

There is much to learn about the full extent of butyrate’s anticancer cell potential. Our study, for instance, concentrated solely on the impact of butyrate on the ERK1/2-c-Myc pathway and cancer cell proliferation although there are complex oncogenic signaling networks with multi-pathways involved in cancer cell death and survival. To gain further insights into the inhibitory efficacy of butyrate, future investigations should explore the impact of butyrate on other crucial signaling pathways (e.g., Wnt/β-catenin) in relationship to cancer cell types and human clinical data.

## 5. Conclusions

**Figure 6:**
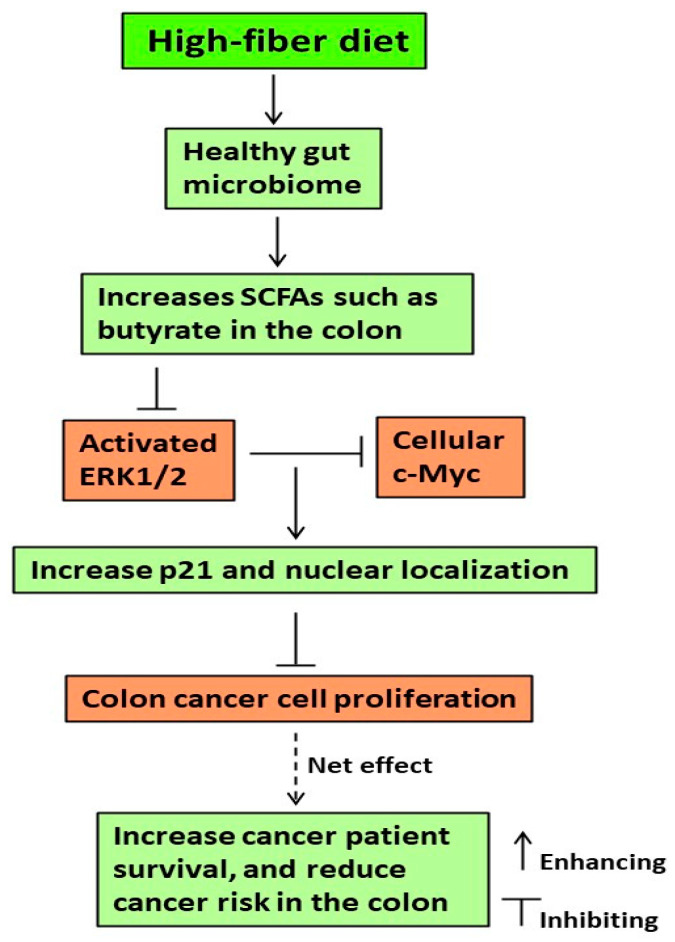
Proposed molecular events underlying the efficacy of diet-related butyrate to inhibit colonic cancer cell growth.

Taken together, although a high-fiber diet is acknowledged for promoting a healthy gut microbiome and producing SCFAs like butyrate [9,16], our study reveals that butyrate effectively inhibits colon cancer cell growth through the ERK1/2-c-Myc-p21 pathway. Importantly, this inhibitory efficacy is cell type-specific and apoptosis-dependent (Figure 6). Moreover, at the molecular and cellular level, our findings provide a novel approach to understanding the clinical data in which the 5-year survival time (after diagnosis) of the high p21 expression colon cancer cohort is greater than that of the low p21 expression colon cancer cohorts (Figure 6).

## Data Availability

The data in this study can be obtained upon request from the corresponding author.

## Supplementary Materials

The following supporting information can be downloaded at: https://www.mdpi.com/article/10.3390/nu16040529/s1, Figure S1: The corresponding dot plot images; Figure S2: The corresponding p21 protein (green signals) and PI cell background specifically nuclei (red signals) images.

## References

[1] Siegel R.L., Wagle N.S., Cercek A., Smith R.A., Jemal A. (2023). Colorectal cancer statistics, 2023. CA Cancer J. Clin..

[2] Anand P., Kunnumakkara A.B., Sundaram C., Harikumar K.B., Tharakan S.T., Lai O.S., Sung B., Aggarwal B.B. (2008). Cancer is a preventable disease that requires major lifestyle changes. Pharm. Res..

[3] Satia J.A., Keku T., Galanko J.A., Martin C., Doctolero R.T., Tajima A., Sandler R.S., Carethers J.M. (2005). Diet, lifestyle, and genomic instability in the North Carolina Colon Cancer Study. Cancer Epidemiol. Biomark. Prev..

[4] Perrin P., Pierre F., Patry Y., Champ M., Berreur M., Pradal G., Bornet F., Meflah K., Menanteau J. (2001). Only fibres promoting a stable butyrate producing colonic ecosystem decrease the rate of aberrant crypt foci in rats. Gut.

[5] Reddy B.S., Hirose Y., Cohen L.A., Simi B., Cooma I., Rao C.V. (2000). Preventive potential of wheat bran fractions against experimental colon carcinogenesis: Implications for human colon cancer prevention. Cancer Res..

[6] Slavin J.L. (2008). Position of the American Dietetic Association: Health implications of dietary fiber. J. Am. Diet. Assoc..

[7] Papathanasopoulos A., Camilleri M. (2010). Dietary fiber supplements: Effects in obesity and metabolic syndrome and relationship to gastrointestinal functions. Gastroenterology.

[8] Raninen K., Lappi J., Mykkanen H., Poutanen K. (2011). Dietary fiber type reflects physiological functionality: Comparison of grain fiber, inulin, and polydextrose. Nutr. Rev..

[9] Zeng H., Lazarova D.L., Bordonaro M. (2014). Mechanisms linking dietary fiber, gut microbiota and colon cancer prevention. World J. Gastrointest. Oncol..

[10] Fleming S.E., Marthinsen D., Kuhnlein H. (1983). Colonic function and fermentation in men consuming high fiber diets. J. Nutr..

[11] Fleming S.E., O’Donnell A.U., Perman J.A. (1985). Influence of frequent and long-term bean consumption on colonic function and fermentation. Am. J. Clin. Nutr..

[12] Cummings J.H., Pomare E.W., Branch W.J., Naylor C.P., Macfarlane G.T. (1987). Short chain fatty acids in human large intestine, portal, hepatic and venous blood. Gut.

[13] Degirolamo C., Modica S., Palasciano G., Moschetta A. (2011). Bile acids and colon cancer: Solving the puzzle with nuclear receptors. Trends Mol. Med..

[14] Intlekofer A.M., Finley L.W.S. (2019). Metabolic signatures of cancer cells and stem cells. Nat. Metab..

[15] Yen T.H., Wright N.A. (2006). The gastrointestinal tract stem cell niche. Stem Cell Rev..

[16] Zeng H., Umar S., Rust B., Lazarova D., Bordonaro M. (2019). Secondary Bile Acids and Short Chain Fatty Acids in the Colon: A Focus on Colonic Microbiome, Cell Proliferation, Inflammation, and Cancer. Int. J. Mol. Sci..

[17] Zeng H., Hamlin S.K., Safratowich B.D., Cheng W.H., Johnson L.K. (2020). Superior inhibitory efficacy of butyrate over propionate and acetate against human colon cancer cell proliferation via cell cycle arrest and apoptosis: Linking dietary fiber to cancer prevention. Nutr. Res..

[18] Lannagan T.R., Jackstadt R., Leedham S.J., Sansom O.J. (2021). Advances in colon cancer research: In vitro and animal models. Curr. Opin. Genet. Dev..

[19] National Cancer Institue (2024). The Cancer Genome Atlas Program. https://www.cancer.gov/ccg/research/genome-sequencing/tcga.

[20] Yeung T.M., Gandhi S.C., Wilding J.L., Muschel R., Bodmer W.F. (2010). Cancer stem cells from colorectal cancer-derived cell lines. Proc. Natl. Acad. Sci. USA.

[21] Lea T., Verhoeckx K., Cotter P., López-Expósito I., Kleiveland C., Lea T., Mackie A., Requena T., Swiatecka D., Wichers H. (2015). Caco-2 Cell Line. The Impact of Food Bioactives on Health: In Vitro and Ex Vivo Models.

[22] Yang B., Cao L., Liu B., McCaig C.D., Pu J. (2013). The transition from proliferation to differentiation in colorectal cancer is regulated by the calcium activated chloride channel A1. PLoS ONE.

[23] Monittola F., Bianchi M., Nasoni M.G., Luchetti F., Magnani M., Crinelli R. (2023). Gastric cancer cell types display distinct proteasome/immunoproteasome patterns associated with migration and resistance to proteasome inhibitors. J. Cancer Res. Clin. Oncol..

[24] Luo Q., Zhou P., Chang S., Huang Z., Zeng X. (2023). Characterization of butyrate-metabolism in colorectal cancer to guide clinical treatment. Sci. Rep..

[25] Li J., Ma X., Chakravarti D., Shalapour S., DePinho R.A. (2021). Genetic and biological hallmarks of colorectal cancer. Genes. Dev..

[26] Louis K.S., Siegel A.C. (2011). Cell viability analysis using trypan blue: Manual and automated methods. Methods Mol. Biol..

[27] Chen T.R. (1977). In situ detection of mycoplasma contamination in cell cultures by fluorescent Hoechst 33,258 stain. Exp. Cell Res..

[28] Rahib L., Wehner M.R., Matrisian L.M., Nead K.T. (2021). Estimated Projection of US Cancer Incidence and Death to 2040. JAMA Netw. Open.

[29] Vedenov D., Pesti G.M. (2008). A comparison of methods of fitting several models to nutritional response data. J. Anim. Sci..

[30] Fan J., Wray J., Meng X., Shen Z. (2009). BCCIP is required for the nuclear localization of the p21 protein. Cell Cycle.

[31] de Visser K.E., Joyce J.A. (2023). The evolving tumor microenvironment: From cancer initiation to metastatic outgrowth. Cancer Cell.

[32] Greaves M. (2015). Evolutionary determinants of cancer. Cancer Discov..

[33] Hinnebusch B.F., Meng S., Wu J.T., Archer S.Y., Hodin R.A. (2002). The effects of short-chain fatty acids on human colon cancer cell phenotype are associated with histone hyperacetylation. J. Nutr..

[34] Scharlau D., Borowicki A., Habermann N., Hofmann T., Klenow S., Miene C., Munjal U., Stein K., Glei M. (2009). Mechanisms of primary cancer prevention by butyrate and other products formed during gut flora-mediated fermentation of dietary fibre. Mutat. Res..

[35] Schlörmann W., Horlebein C., Hübner S.M., Wittwer E., Glei M. (2023). Potential Role of ROS in Butyrate- and Dietary Fiber-Mediated Growth Inhibition and Modulation of Cell Cycle-, Apoptosis- and Antioxidant-Relevant Proteins in LT97 Colon Adenoma and HT29 Colon Carcinoma Cells. Cancers.

[36] Bultman S.J. (2014). Molecular pathways: Gene-environment interactions regulating dietary fiber induction of proliferation and apoptosis via butyrate for cancer prevention. Clin. Cancer Res..

[37] Kaźmierczak-Siedlecka K., Marano L., Merola E., Roviello F., Połom K. (2022). Sodium butyrate in both prevention and supportive treatment of colorectal cancer. Front. Cell. Infect. Microbiol..

[38] Zeng H., Briske-Anderson M. (2005). Prolonged butyrate treatment inhibits the migration and invasion potential of HT1080 tumor cells. J. Nutr..

[39] Lu Z., Xu S. (2006). ERK1/2 MAP kinases in cell survival and apoptosis. IUBMB Life.

[40] Sun Y., Liu W.Z., Liu T., Feng X., Yang N., Zhou H. (2015). Signaling pathway of MAPK/ERK in cell proliferation, differentiation, migration, senescence and apoptosis. J. Recept. Signal Transduct. Res..

[41] Ullah R., Yin Q., Snell A.H., Wan L. (2022). RAF-MEK-ERK pathway in cancer evolution and treatment. Semin. Cancer Biol..

[42] Zuo Z., Liu J., Sun Z., Cheng Y.W., Ewing M., Bugge T.H., Finkel T., Leppla S.H., Liu S. (2023). ERK and c-Myc signaling in host-derived tumor endothelial cells is essential for solid tumor growth. Proc. Natl. Acad. Sci. USA.

[43] Tan L., Peng D., Cheng Y. (2022). Significant position of C-myc in colorectal cancer: A promising therapeutic target. Clin. Transl. Oncol..

[44] Augenlicht L.H., Wadler S., Corner G., Richards C., Ryan L., Multani A.S., Pathak S., Benson A., Haller D., Heerdt B.G. (1997). Low-level c-myc amplification in human colonic carcinoma cell lines and tumors: A frequent, p53-independent mutation associated with improved outcome in a randomized multi-institutional trial. Cancer Res..

[45] Wang Z., Liu M., Zhu H., Zhang W., He S., Hu C., Quan L., Bai J., Xu N. (2010). Suppression of p21 by c-Myc through members of miR-17 family at the post-transcriptional level. Int. J. Oncol..

[46] Gartel A.L., Ye X., Goufman E., Shianov P., Hay N., Najmabadi F., Tyner A.L. (2001). Myc represses the p21(WAF1/CIP1) promoter and interacts with Sp1/Sp3. Proc. Natl. Acad. Sci. USA.

[47] Kreis N.N., Louwen F., Yuan J. (2019). The Multifaceted p21 (Cip1/Waf1/CDKN1A) in Cell Differentiation, Migration and Cancer Therapy. Cancers.

[48] Karimian A., Ahmadi Y., Yousefi B. (2016). Multiple functions of p21 in cell cycle, apoptosis and transcriptional regulation after DNA damage. DNA Repair.

[49] Acquaviva R., Tomasello B., Di Giacomo C., Santangelo R., La Mantia A., Naletova I., Sarpietro M.G., Castelli F., Malfa G.A. (2021). Protocatechuic Acid, a Simple Plant Secondary Metabolite, Induced Apoptosis by Promoting Oxidative Stress through HO-1 Downregulation and p21 Upregulation in Colon Cancer Cells. Biomolecules.

[50] Parveen A., Akash M.S., Rehman K., Kyunn W.W. (2016). Dual Role of p21 in the Progression of Cancer and Its Treatment. Crit. Rev. Eukaryot. Gene Expr..

