# Supplementary material for: Efficacy of Butyrate to Inhibit Colonic Cancer Cell Growth Is Cell Type-Specific and Apoptosis-Dependent
Source: Nutrients. 2024 Feb 14;16(4):529. doi: 10.3390/nu16040529 (PMC10892417; doi:10.3390/nu16040529)
Supplement: Supplementary file 1 [file nutrients-16-00529-s001.zip › nutrients-2800287-supplementary.pdf]

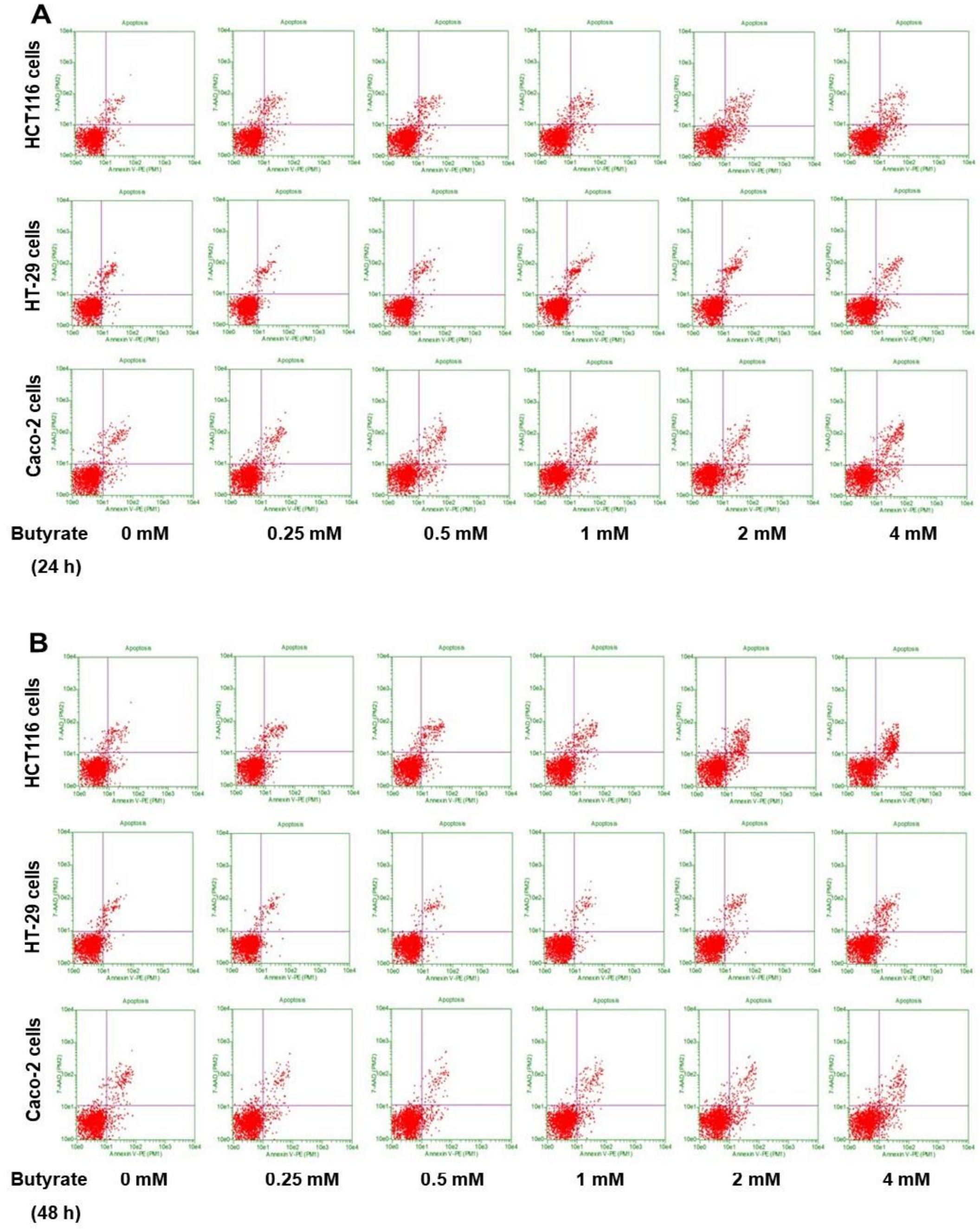

Figure S1. The corresponding (representative) dot plot images of Figure 2. The panel A, and B represented 24 h and 48 h butyrate treatments, respectively.

Events in each of the four quadrants are as follows: (1) upper-left quadrant: mostly nuclear debris [Annexin V-PE(-) and 7-ADD(+)]; (2) upper-right quadrant: cells in the late stages of apoptosis or dead cells (by necrotic or apoptotic mechanisms) [Annexin V-PE(+) and 7-AAD(+)]; (3) lower-left quadrant: viable cells, not undergoing detectable apoptosis [Annexin V-PE(-) and 7-AAD(-)]; (4) lower-right quadrant: cells in the early to mid-stages of apoptosis [Annexin V-PE(+) and 7-AAD(-)].

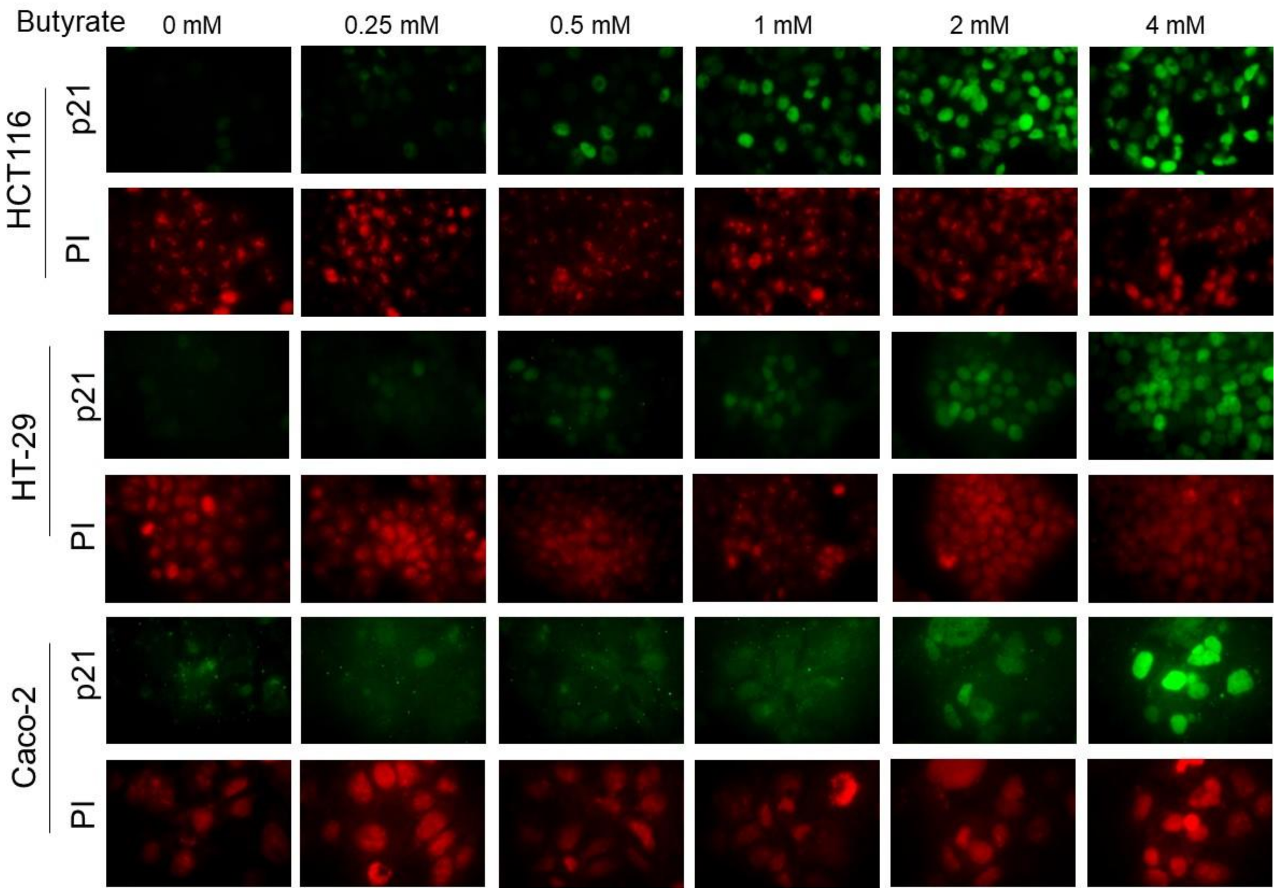

Figure S2. The corresponding p21 protein (green signals) and PI cell background specifically nuclei (red signals), individually, for merged images of Figure 4.
